# Diagnostic Accuracy of Apple Watch Electrocardiogram for Atrial Fibrillation: A Systematic Review and Meta-Analysis

**DOI:** 10.1016/j.jacadv.2024.101538

**Published:** 2025-01-09

**Authors:** Sufyan Shahid, Minahil Iqbal, Humza Saeed, Sara Hira, Amna Batool, Salman Khalid, Naeem Khan Tahirkheli

**Affiliations:** a Department of Cardiology, Khawaja Muhammad Safdar Medical College, Sialkot, Pakistan; b Department of Cardiology, Allama Iqbal Medical College, Lahore, Pakistan; c Department of Cardiology, Rawalpindi Medical University, Rawalpindi, Pakistan; d Department of Cardiology, Fatima Memorial Hospital, Lahore, Pakistan; e Department of Cardiology, Oklahoma Heart Hospital, Oklahoma, USA

**Keywords:** Apple Watch, electrocardiogram, atrial fibrillation, heart rhythm, atrial fibrillation screening

## Abstract

**Background:**

Electrocardiography (ECG) is the gold standard for the diagnosis of atrial fibrillation (AF). Recently, smartwatches like the Apple Watch have emerged as a promising, user-friendly device for rapid detection and diagnosis of AF, but the reliability and diagnostic accuracy still remain controversial.

**Objectives:**

The purpose of this study was to perform a systematic review and diagnostic test accuracy meta-analysis evaluating the diagnostic performance of the Apple Watch ECG in detecting AF.

**Methods:**

The literature search was conducted on PubMed, Embase, and Cochrane Library through April 2024 for studies comparing the diagnostic accuracy of Apple Watch to standard 12-lead ECG. Statistical analysis was performed using R Software version 4.4.0 and OpenMeta[Analyst]. Pooled analyses of sensitivity, specificity, and area under the receiver operating characteristic curve were determined along with their 95% CIs. The quality of studies was analyzed using the QUADAS-2 tool.

**Results:**

The meta-analysis included 11 studies comprising 4,241 participants. Their mean age was 62.56 ± 3.92 years, and 28% of the patients were females. The pooled sensitivity and specificity of the Apple Watch for detecting AF were 94.8% (95% CI: 91.7% to 96.8%; I^2^ = 67%) and 95% (95% CI: 88.6% to 97.8%; I^2^ = 88%), respectively. The area under the receiver operating characteristic curve was 0.96 (95% CI: 0.92-0.97).

**Conclusions:**

The Apple Watch ECG carries high accuracy in detecting atrial fibrillation, providing a convenient diagnostic option for patients.

Atrial fibrillation (AF) is a prevalent cardiac arrhythmia characterized by an irregular rhythm, which is evident on an electrocardiogram (ECG) due to the loss of coordinated atrial contractility. The risk of developing AF is around 37% after 55 years of age.^1^ AF usually presents with symptoms like palpitations, fatigue, lightheadedness, and fainting and can also be asymptomatic. The absence of specific symptoms in many patients can lead to delayed diagnosis, potentially resulting in clinical consequences, with stroke often being the first noticeable presentation. Timely diagnosis leading to early antithrombotic treatment can prevent this lethal complication. ECG has been used as the gold standard for diagnosing AF since the early 1900s.^2^

In today’s world, advancements in technology have made smartwatches a crucial part of everyday life. In the United States, 13% of people own smartwatches, with an additional 40% showing interest in buying 1. The use of smart devices has surged from 325 million connected devices in 2016 to 1.1 billion worldwide by 2022.^3^ Apple Watch stands out as the most popular among smartwatches, becoming the first to receive Food and Drug Administration (FDA) approval for single-lead ECG monitoring. Early-stage diagnoses have become more common as more consumers use these smartwatches that can self-record and auto-diagnose heart rhythm disorders like AF. This technological breakthrough allows for the noninvasive and user-friendly monitoring of vital cardiac parameters such as heart rate and rhythm and can improve the delivery of health care with portable ECG devices.

The Apple Watch can detect AF by continuously monitoring heart rate and its variability using an optical photo-plethysmo-graphic (PPG) sensor located on the underside of the watch.^4^ To capture an ECG reading, the individual puts the watch on their left wrist, accesses the ECG application, and gently places a finger from their right hand on the digital crown. The watch then initiates a 30-second recording session, replicating a conventional lead-I ECG by creating a bipolar signal derived from the voltage variations between the left and right arms. These devices also have a built-in algorithm to classify the rhythm strip as AF, sinus rhythm, or inconclusive.^5^ However, the data on the diagnostic accuracy of the Apple Watch is scarce. Previous meta-analyses by Belani et al^6^ and Prasitlumkum et al^7^ looked into various wearable devices and were not specific to the Apple Watch but were limited by the number of studies and sample size.

American College of Cardiology (ACC) and European Society of Cardiology (ESC) recent guidelines suggest using wearable devices for diagnosis and long-term surveillance of AF but suggest caution in use due to lack of clinical validation.^8^^,^^9^ The Apple Watch, being a low-risk device, has received FDA clearance but did not go through a formal FDA approval process, which requires extensive testing. Therefore, through this systematic review and diagnostic test accuracy (DTA) meta-analysis, we aim to evaluate the diagnostic accuracy of the Apple Watch in detecting atrial fibrillation by comparing it with standard 12 lead-ECG. We hypothesize that the Apple Watch has a comparable diagnosing accuracy to a standard 12-lead ECG.

## Methods

### Literature review and search strategy

This systematic review and DTA meta-analysis followed the Preferred Reporting Items for Systematic Reviews and Meta-Analysis of DTA studies guidelines.^10^ The study protocol is available at the International Prospective Register of Systematic Reviews (PROSPERO) under the identifier CRD42024530407.

A systematic literature search of Medline/PubMed (Ovid), Embase (Ovid), and the Cochrane Central Register of Controlled Trial was conducted from their inception to March 25, 2024, to identify relevant original full-length articles without any language restrictions. The search was done by database-specific Boolean search strategies, using the following terms: (“apple smartwatch” OR “apple watch” OR “watch”) AND (“ECG” OR “electrocardiogram” OR “EKG” OR “Electrocardiography”) AND (“atrial fibrillation” OR “atrial arrhythmias”). A manual search for additional relevant studies using references found in the included articles was also performed.

### Eligibility criteria

Studies were included in this meta-analysis if they involved human subjects, evaluated the accuracy of Apple smartwatch against 12-lead standard ECG, diagnosed atrial fibrillation, and provided effect estimates of overall sensitivity (%) and specificity (%) with 95% CIs. The exclusion criteria included abstracts without full-length articles, unpublished studies, notes, letters, comments, conference articles, and studies lacking ECG monitoring or having overlapping patient populations in multiple studies. The articles retrieved from the systematic search were exported to the EndNote version 21.0, where duplicates were screened for and removed. Two authors blindly reviewed the titles and abstracts to check the study's eligibility according to the inclusion and exclusion criteria, and if both agreed, the study was eligible for full-text review. Disagreements were solved by consensus or referred to the third author.

### Data extraction

Of the included studies in the review, 2 researchers performed data extraction independently. The data collected included the study author’s name, the year of publication, type of device used, sample size, number of recorded events, method of AF verification, true positive, true negative, false positive, false negative, and effect estimates of overall sensitivity (%) and specificity (%) with 95% CIs.

### Data synthesis

We used a bivariate random-effects model to pool sensitivity and specificity estimates across studies, taking into account the potential heterogeneity and correlation between sensitivity and specificity within each study. MetaDTA software (version 2.0.5) was used for analysis since it implements the bivariate method and is specifically designed for diagnostic meta-analyses. The pooled estimates of sensitivity and specificity across studies were visually represented by forest plots.

### Statistical analysis

Statistical analysis was performed using R for Windows (version 4.4.0) and OpenMeta[Analyst]. The MADA R package was utilized to compute pooled sensitivity and specificity and to create summary receiver operating characteristic (SROC) curves. Adjusted point estimates from each study were combined using the generic inverse variance method of Der Simonian and Laird, which assigns weights to each study based on their variance. Due to the potential for increased interobservation variance, a random-effects model was employed to evaluate (with 95% CIs) the pooled sensitivity and specificity of Apple devices. Cochran's Q test and I2 statistics were applied to assess between-study heterogeneity, with I2 values categorized as follows: 0 to 25% indicating insignificant heterogeneity, 26% to 50% indicating low heterogeneity, 51% to 75% indicating moderate heterogeneity, and >75% indicating high heterogeneity. A bivariate random-effects regression model was used for pooling sensitivity and specificity, and SROC curves were generated based on this model. A sensitivity analysis was conducted to evaluate the impact of individual studies on the overall results by categorizing studies based on patient characteristics at the time of study enrollment, specifically the presence or absence of a history of atrial fibrillation (AF).

### Quality assessment

The Quality Assessment of Diagnostic Accuracy Studies (QUADAS-2) tool was used to evaluate the methodological quality and potential sources of bias in each study. The assessment focused on 4 crucial domains: patient selection, index test, reference standard, and flow and timing (which encompasses patient progression throughout the study and the timing of the index test and reference standard). For each domain, the risk of bias was classified as low, high, or unclear. Additionally, concerns regarding the applicability of the findings were assessed for the patient selection, index test, and reference standard domains.

## Results

### Search results

**Figure 1:**
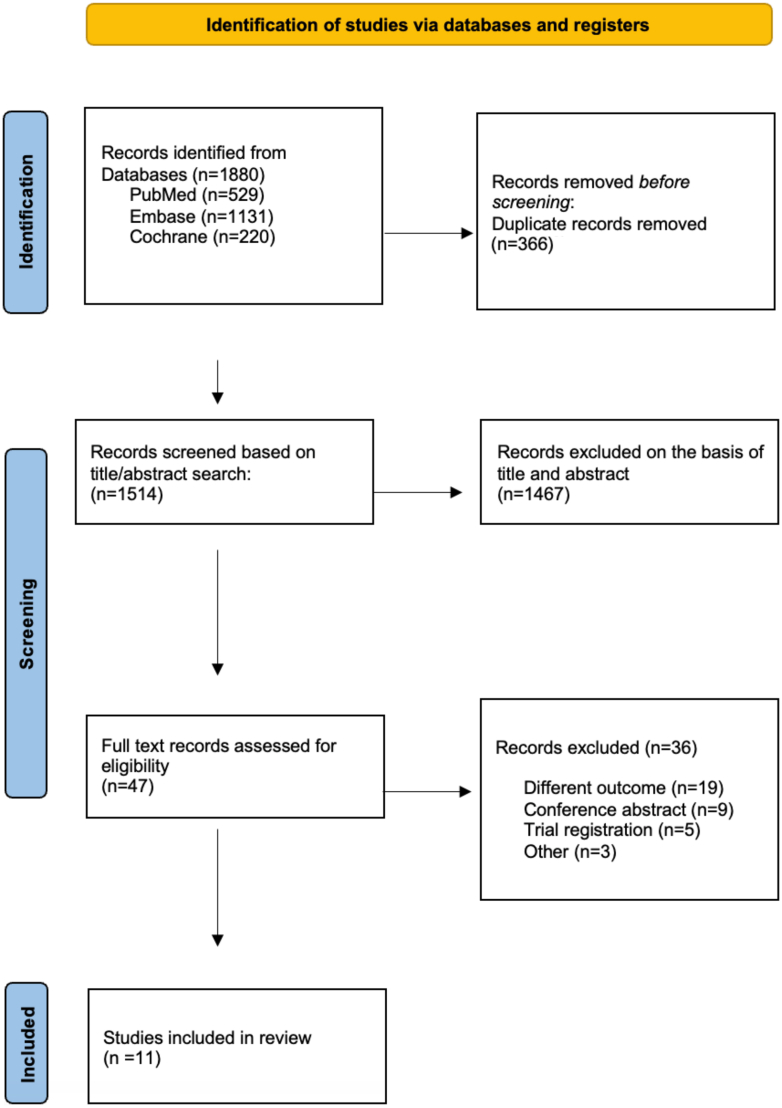
**PRISMA Flow Diagram of the Included Studies** PRISMA = Preferred Reporting Items for Systematic Reviews and Meta-Analysis.

Our search strategy yielded a total of 1,880 articles. After removing duplicates, 1,514 studies were screened by title and abstract, resulting in the exclusion of 1,467 studies. Subsequent full-text review of the remaining 47 studies led to the exclusion of 36 studies that did not meet the inclusion criteria. Ultimately, 11 studies^11^, ^12^, ^13^, ^14^, ^15^, ^16^, ^17^, ^18^, ^19^, ^20^ with 13,940 patients were included in this meta-analysis. The literature retrieval, review, and selection processes are shown in Figure 1.

### Study characteristics

A total of 4,241 participants from 11 studies were included, each conducted in a different clinical setting with varied participant demographics. All studies used single-lead Apple Watch ECG outputs as the index test and 12-lead ECG as the reference standard. PPG was the common software in different series of Apple Watches, as assessed by all studies. Three studies included patients with a history of AF only, and the rest had patients irrespective of AF history. The mean age of the participants from the studies was 62.56 ± 3.92 years. The percentage of females across all studies was 28%. The overall prevalence of AF was found to be 47%. The percentage of inconclusive tracings has been added in Supplementary Table 1. The general characteristics of the included studies, including the index tests and reference standards employed, demographics of the participants, and type of Apple Watch used, are presented in Table 1.

**Table 1:** Baseline Characteristics of Included Studies

First Author	Year	Study Design	Country	Sample Size	Participants	Prevalence of AF %	Male/Female, %	Mean Age, y	Reference Standard	Watch	Sensitivity	Sensitivity
Paslı	2024	Prospective observational study	Turkey	721	Patients presenting to ED with severe but not immediately life threatening as well as life-threatening conditions requiring immediate care.	25	54/46	66.5 ± 17.1	Standard 12-lead ECGs	Apple Watch series 7	96	100
Mannhart	2023	Prospective diagnostic study	Switzerland	201	Patients scheduled for catheter ablation procedures, electric cardioversions, pacemaker, or implantable cardioverter-defibrillator implantation were included	31	69/31	67	12-lead ECG	Apple Watch Series 6	93	96
Velraeds	2023	nr	France	723	Patients hospitalized in cardiology department; 21% had AF, 24% had an ECG without any abnormality (no disease)	21	nr	nr	12-lead ECG	Apple Watch Series 5	90	92
Alnasser	2023	nr	Saudi Arabia	469	Patients presenting to outpatient cardiac clinics both with cardiac and other diseases.	7	56.3/43.7	56.43 ± 16.3	12-lead ECG	Apple Watch Series 6	81.08	99.54
Scholten	2020	Prospective, nonrandomized, adjudicator-blinded study	Netherlands	220	Adult patients admitted for electrical cardioversion (ECV) for AF or atrial flutter (AFL)	100	65/35	70 ± 10	12-lead ECG	Apple Watch series 5	93	96
Ploux	2022	Prospective observational study	France	260	Patients with or without a history of cardiovascular disease who presented to our hospital outpatient and emergency departments	19	58/42	66 ± 6	12-lead ECG	Apple watch Series 4	96	91
Pepplinkhuizen	2022	Prospective, nonrandomized, single-center observational study	Netherlands	74	18 years or older patients scheduled for elective electrical cardioversion (ECV)	100	79.7/20.3	67.1 ± 12.3	12-lead ECG	Apple watch series 6	93.5	100
Abu-Alrub	2022	Prospective, nonrandomized, adjudicator-blinded study	Germany	200	Patients in sinus rhythm who had undergone an AF ablation procedure in the previous 6 months and patients in persistent or permanent AF who were referred for catheter ablation. All patients were ≥18 years of age	100	56/44	62 ± 7	12-lead ECG	Apple Watch Series 5	90	95
Racine	2022	Prospective nonrandomized study	France	734	Hospitalized patients ≥18 years of age.	21	58/42	66	12-lead ECG	Apple Watch Series 5	97	88
Apple	2018	Prospective single-arm pragmatic study	USA	588	Study participants with known AF and others with no known cardiac rhythm abnormalities were enrolled.	51	N/A	nr	12-lead ECG	Apple Watch Series 4, 5, 6	98.3	99
Tison	2018	Retrospective Cohort study	USA	51	Patients with AF presenting for electrical cardioversion or pharmacologic cardioversion	100	84/16	66.1 ± 10.7	12-lead ECG	Apple Watch Series 4 (Cardiogram application)	98	90

AF = atrial fibrillation; ECG = electrocardiogram; nr = not reported.

### Diagnostic accuracy of Apple Watch for the presence of AF

**Figure 2:**
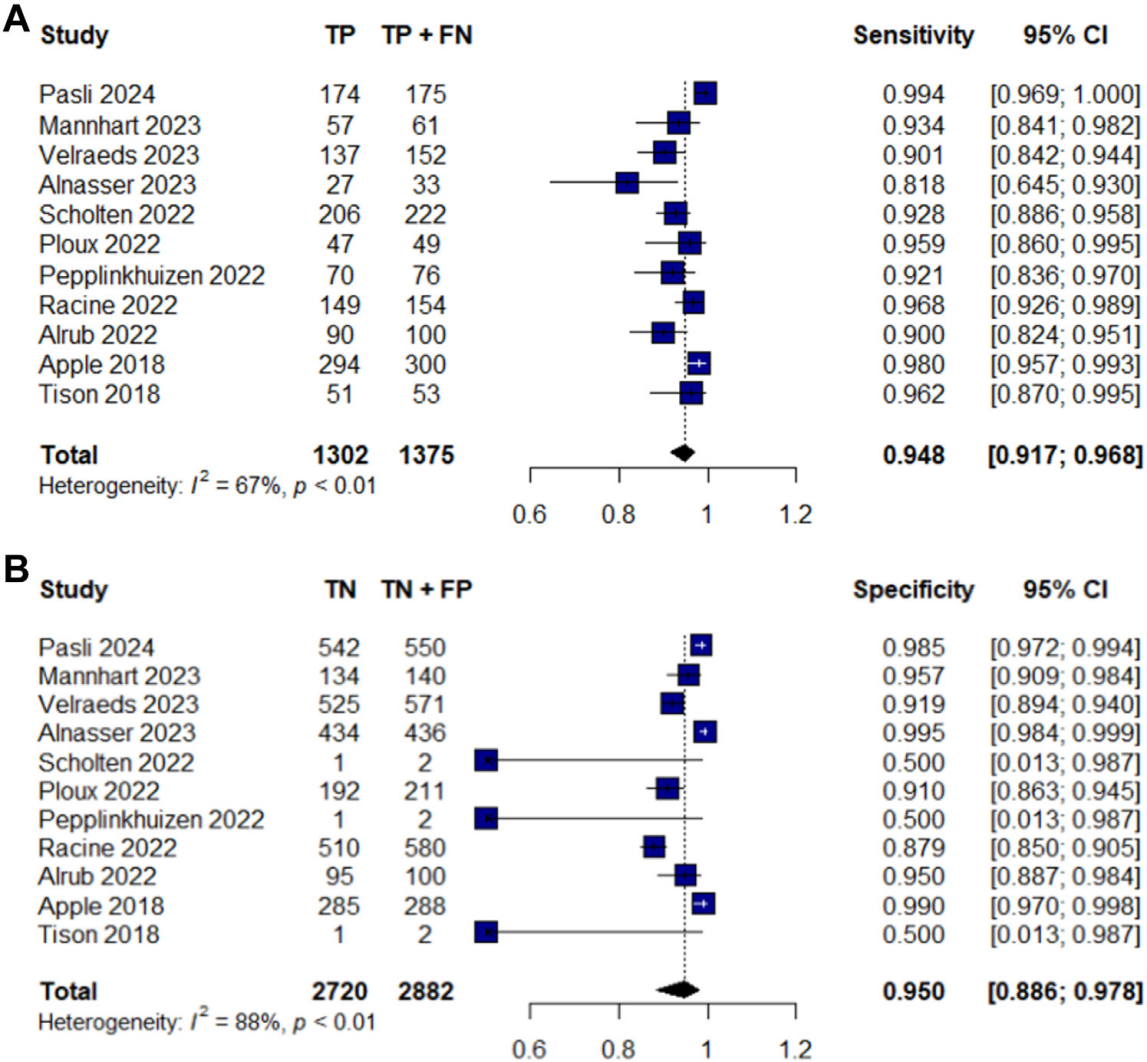
Forest Plots for the Pooled Sensitivity and Specificity of the Studies (A) Forest plots for pooled sensitivity of the studies. Horizontal lines represent 95% CIs of the individual studies. (B) Forest plots for pooled specificity of the studies. Horizontal lines represent 95% CIs of the individual studies. TN = true negative; FP = false positive; TP = true positive; FN = false negative.

The pooled sensitivity and specificity of Apple Watch ECG for detecting AF were 94.8% (95% CI: 91.7% to 96.8%) and 95% (95% CI: 88.6% to 97.8%), respectively. Forest plots for sensitivity and specificity are included in Figures 2A and 2B to visually represent these findings.

**Figure 3:**
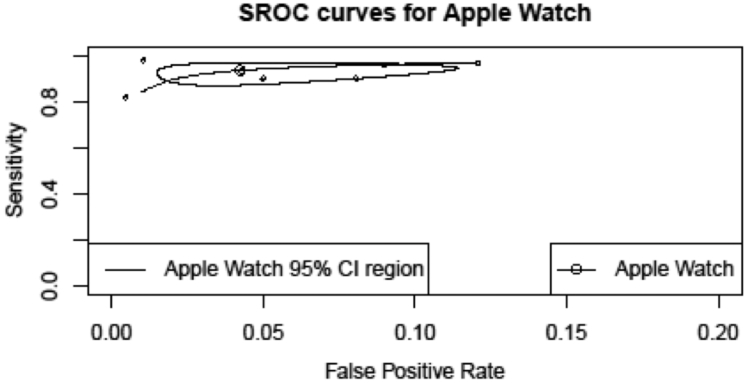
**Hierarchical Summary Receiver Operating Characteristic Curve** The “data” points show performance for each study, and the “summary estimate” points represent the pooled performance. SROC = summary receiver operating characteristic.

The hierarchical SROC curve is shown in Figure 3 with the area under the curve of 0.96 (95% CI: 0.92-0.97). The pooled diagnostic odds ratio of the studies was 346.464 (95% CI: 123.769-969.846) and pooled likelihood ratio positive and likelihood ratio negative were found to be 18.849 (95% CI: 8.332-42.642) and 0.054 (95% CI: 0.034-0.087), respectively.

### Heterogeneity assessment

According to the Higgins' I^2^ statistics, both sensitivity and specificity show remarkable heterogeneity between the studies (I^2^ = 67% for sensitivity and I^2^ = 88% for specificity).

### Quality assessment

Based on the QUADAS-2 tool, 7 studies (63.6%) demonstrated a high risk of bias in patient selection and flow and timing. Additionally, 6 studies (54.5%) exhibited high applicability concerns regarding patient selection. The individual assessments of risk of bias and applicability concerns for each study are detailed in the Supplemental Table 2.

### Subgroup analysis

**Figure 4:**
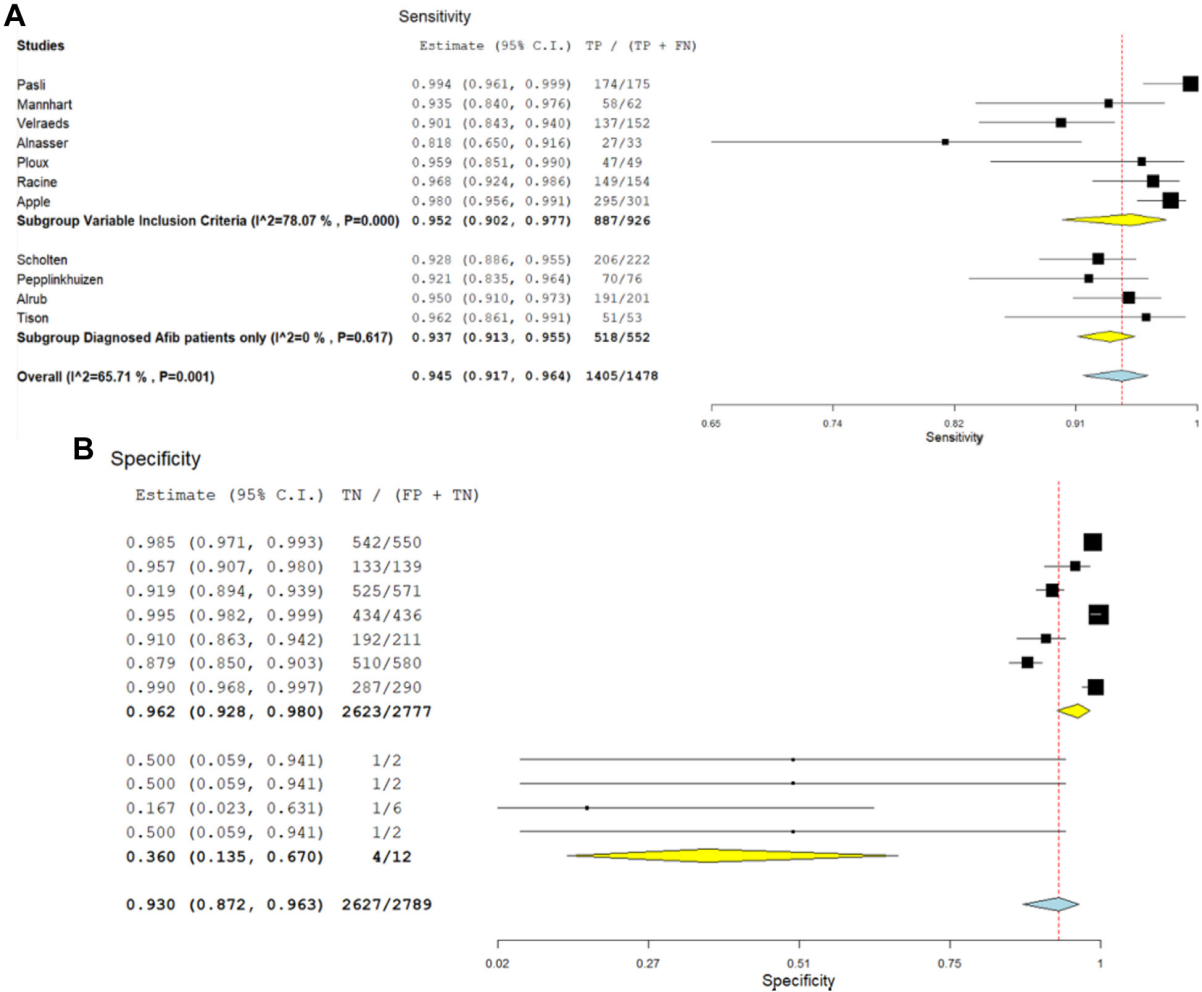
Subgroup Analysis (A) Subgroup analysis of studies for sensitivity based on inclusion of AF participants. Horizontal lines represent 95% CIs of the individual studies. (B) Subgroup analysis for specificity based on inclusion criteria of studies. Horizontal lines represent 95% CIs of the individual studies. TN = true negative; FP = false positive; TP = true positive; FN = false negative.

The subgroup analysis was performed based on the inclusion of studies with participants with a previous history of AF. When studies with participants having a previous history of AF were considered, the subgroup analysis revealed a notable reduction in heterogeneity (to 0%) among studies focusing exclusively on AF patients. In contrast, significant heterogeneity remained among studies with patients of diverse clinical profiles, indicating persistent variability in their findings. The results of this subgroup analysis for sensitivity and specificity are shown in Figures 4A and 4B, respectively.

### Meta regression

**Figure 5:**
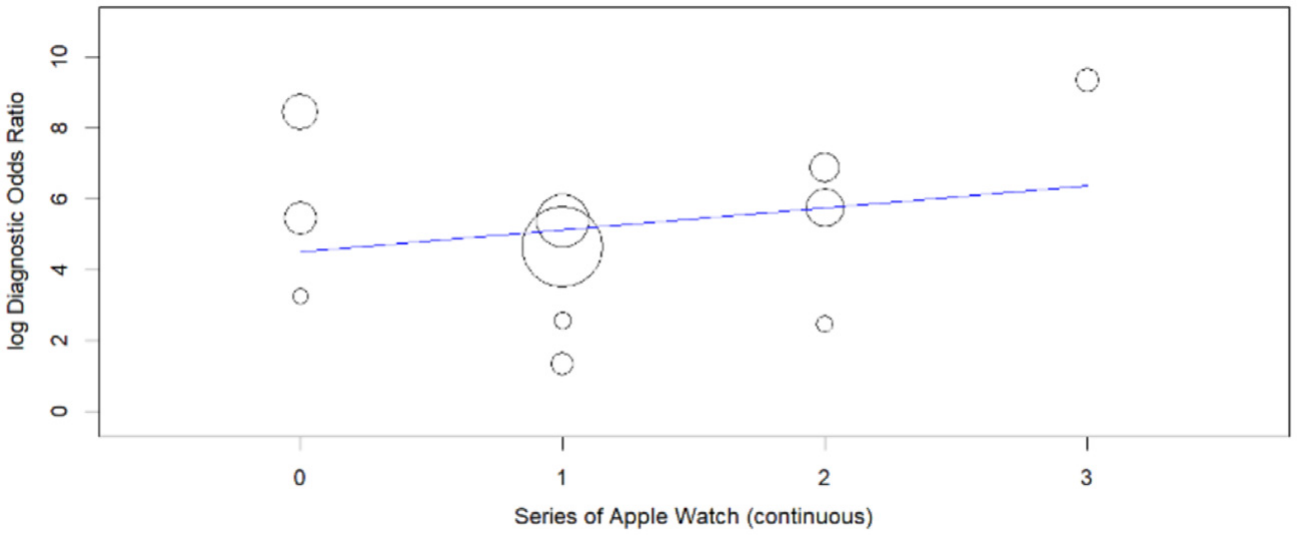
**Meta Regression Plot** The intercept shows the relationship between the series of Apple Watches employed and the resulting diagnostic odds ratio observed.

Meta-regression results showed a linear relationship between diagnostic odds ratio and series of Apple Watches used, indicating that use of upgraded series leads to better odds of diagnosing atrial fibrillation (Figure 5).

## Discussion

This systematic review and meta-analysis found that the Apple Watch ECG has high diagnostic accuracy in detecting AF with a pooled sensitivity of 94.8% and a specificity of 95% when compared with standard 12-lead ECG. The receiver operating characteristic curve's area under the curve was found to be 0.96, indicating excellent diagnostic performance. In addition, a high diagnostic odds ratio, a high positive likelihood ratio, and a low negative likelihood ratio further validated the diagnostic accuracy (Central Illustration). Our results are consistent with Belani et al.^6^ (96.99% sensitivity, 95% CI: 95.77-98.20) for wrist-worn AF detection. However, our meta-analysis differs in scope, focusing solely on Apple Watch ECG, using 12-lead ECG as reference, and including patients regardless of AF history. Similarly, Prasitlumkum et al reported high sensitivity and specificity for AF detection using smartwatches and smartphones, highlighting limitations of traditional methods like Holter monitoring and ECG patches. Recent guidelines from ACC/American Heart Association/American College of Chest Physicians/Heart Rhythm Society (2023)^8^ and ESC (2020)^9^ recommend using consumer-accessible electrocardiographic devices (eg, smartwatches) for AF recurrence monitoring. ESC guidelines also encourage artificial intelligence-driven AF detection advancements but caution against integrating unvalidated mobile health technologies and health applications into clinical practice.

**Central Illustration:**
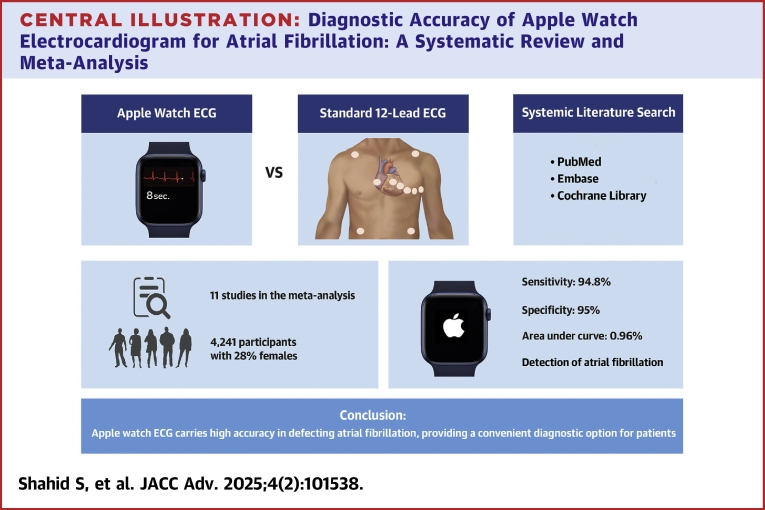
**Diagnostic Accuracy of Apple Watch Electrocardiogram for Atrial Fibrillation: A Systematic Review and Meta-Analysis** ECG = electrocardiogram.

In addition to AF, the Apple Watch is currently being tested in multiple other physiologic scenarios like ECG recording of neonates,^21^ stress prediction,^22^ estimating sleep and heart rate variability,^23^ and for adults with congenital heart disease. This suggests that the Apple Watch can be a useful tool for monitoring heart rhythm in individuals with congenital heart disease, in addition to healthy individuals.^24^ Despite the promising results of our meta-analysis, there is significant heterogeneity among the studies, with I^2^ values of 67% for sensitivity and 88% for specificity, suggesting variability in study outcomes. A significant reduction in heterogeneity to 0% in the subgroup of studies with patients with AF only found no significant differences (heterogeneity) in the results, demonstrating that the studies in this subgroup were consistent with each other. In contrast, significant heterogeneity persisted in the second subgroup of studies having patients of variable clinical profiles (not just AF), still showed significant heterogeneity in their results, showing inconsistency and variability among studies.

Apple Watch Series 4 was used by 3 studies, Series 5 by 4, Series 6 by 3, and Series 7 by only 1 study. Even though various studies utilized different Apple Watch series, all Apple Watch ECG series utilize the same automated algorithms that harness PPG to detect AF by analyzing irregular pulse patterns. Our meta-regression results showed increasing diagnostic accuracy with the use of the advanced Apple Watch series, showing consistent improvement in the algorithms being used. The analyzed studies lacked participant and methodological homogeneity. The included studies had varying patient inclusion criteria, demographics, comorbidities, and diagnostic values. Moreover, the validity of the study findings may be undermined by 2 significant factors: the researchers' prior awareness of the ECG results, which could have introduced bias into their interpretations, and the absence of blinding in test interpretation, potentially leading to compromised accuracy in the reported sensitivity and specificity values. The studies in our review were all prospective observational in nature and all were published in peer-reviewed journals except for the study by Apple Inc.

Half of the studies reported a high bias in patient selection, mainly due to the case-control study design, where physicians knew that they were dealing with already diagnosed cases of AF. Similarly, some studies had a high risk of bias in the flow and timing domain, which was due to the noninclusion of all patients in the final analysis. Although the time interval between the single-lead Apple Watch ECG and the standard 12-lead ECG was not explicitly stated in most studies, nearly all reported using the Apple Watch ECG just after the 12-lead ECG, with minimal delay, except for 1 study (Mannhart et al, 2023), which did not provide information on timing delay. Thus, the consistency in timing across most studies strengthens the reliability of our findings by reducing potential variability related to test timing in AF detection. However, to overcome the existing biases, future studies should avoid case-control designs to better validate the effectiveness of the Apple Watch in diagnosing previously undiagnosed AF.

Regarding the ECG recording methodology, all studies employed a consistent approach, wherein participants first underwent a conventional 12-lead ECG, followed by a 30-second ECG recording using the Apple Watch, ensuring a standardized comparison between the 2 methods. However, Tison et al implied a slight change in methodology by first using the Apple Watch for at least 20 minutes, and then ECG tracings were interpreted by 2 or more blinded, independent cardiologists. The exception to this was Alnasser et al, where researchers made the diagnosis according to predefined definitions and only consulted the cardiologist in case of disagreement. Only Scholten et al have investigated patients with atrial flutter; further studies are warranted to expand knowledge in this area.

Apple Watches show promise as a user-friendly, noninvasive, and cost-effective tool for early AF detection. However, its practical applicability in high-risk populations, particularly older adults, is a concern. While the risk of developing AF increases with age, exceeding 20% by age 80^1^, the majority of Apple Watch users are between 25 and 34 years old.^25^ Similarly, the average age of participants in our analysis was 62.56 ± 3.92 years. This raises concerns on Apple Watch utility in this demographic and warrants further investigation. Future research should focus on validating the Apple Watch's diagnostic performance in larger, undiagnosed populations, assessing its clinical utility, generalizability, and cost-effectiveness. Moreover, researchers should explore the potential burden on primary care providers due to false positives and associated economic implications. It is also important to note that the Apple Watch ECG received FDA clearance through the 510(k) process,^26^ which differs from full FDA approval and does not require extensive clinical trials. Thus, conducting large-scale trials would provide more robust evidence of its diagnostic reliability.

### Study Limitations

To the best of our knowledge, this systematic review and meta-analysis represents the most comprehensive and up-to-date evaluation of the Apple Watch's diagnostic accuracy for AF. However, our study has several limitations that should be acknowledged. Firstly, the observational design of the included studies may have introduced bias, as residual confounders could not be entirely eliminated, potentially leading to over- or under-estimation of diagnostic accuracy. Secondly, significant heterogeneity was observed, likely due to variations in demographic characteristics, AF history, and other unidentified factors among the included studies. Thirdly, the inclusion of patients with and without a history of AF may have introduced selection bias. To address these limitations, we conducted subgroup and sensitivity analyses, which revealed minimal impacts. Fourthly, while 28% of participants were female, gender-specific diagnostic accuracy results were not reported in the included studies. Consequently, a sensitivity analysis based on gender could not be conducted, limiting our ability to assess whether diagnostic accuracy varied by sex. Fifth, we used the QUADAS-2 tool to assess risk of bias; however, this tool does not evaluate observer-related bias, and the included studies did not report on the number of human observers or inter-/intra-observer variability in diagnosing AF with 12-lead ECG, which may influence diagnostic accuracy. Finally, the relatively small sample sizes may have led to an overestimation of specificity due to a limited number of true negatives and false positives.

## Conclusions

Our systematic review and meta-analysis show that the Apple Watch ECG demonstrates high diagnostic accuracy for detecting AF, with high sensitivity and specificity. However, considerable heterogeneity exists in sensitivity and specificity, suggesting variability among study results. Despite Apple Watch having a high diagnostic accuracy for detecting AF, further studies with better methodology are needed to improve compliance, evaluate treatment rates, clinical implications, generalizability, and cost-effectiveness of Apple Watch to elucidate whether or not Apple Watch ECG is a reliable AF detection modality as compared to standard 12-lead ECG.

## Funding support and author disclosures

The authors have reported that they have no relationships relevant to the contents of this paper to disclose.
